# Non-crystalline light chain proximal tubulopathy, a morphologically protean entity

**DOI:** 10.1093/ndt/gfad085

**Published:** 2023-04-29

**Authors:** Andreas Kousios, Sarah Blakey, Linda Moran, Maria Atta, Rawya Charif, Neill Duncan, Andrew Smith, Frederick W K Tam, Jeremy B Levy, Aristeidis Chaidos, Candice Roufosse

**Affiliations:** Imperial College, Centre for Inflammatory Disease, Dept Immunology and Inflammation, Faculty of Medicine, London, UK; West London Renal and Transplant Centre, Hammersmith Hospital, Imperial College Healthcare NHS Trust, London, UK; Imperial College, Centre for Inflammatory Disease, Dept Immunology and Inflammation, Faculty of Medicine, London, UK; West London Renal and Transplant Centre, Hammersmith Hospital, Imperial College Healthcare NHS Trust, London, UK; North West London Pathology, Charing Cross Hospital, London, UK; Department of Haematology, Hammersmith Hospital, Imperial College Healthcare NHS Trust, London, UK; Imperial College, Centre for Inflammatory Disease, Dept Immunology and Inflammation, Faculty of Medicine, London, UK; Imperial College, Centre for Inflammatory Disease, Dept Immunology and Inflammation, Faculty of Medicine, London, UK; West London Renal and Transplant Centre, Hammersmith Hospital, Imperial College Healthcare NHS Trust, London, UK; North West London Pathology, Charing Cross Hospital, London, UK; Imperial College, Centre for Inflammatory Disease, Dept Immunology and Inflammation, Faculty of Medicine, London, UK; West London Renal and Transplant Centre, Hammersmith Hospital, Imperial College Healthcare NHS Trust, London, UK; Imperial College, Centre for Inflammatory Disease, Dept Immunology and Inflammation, Faculty of Medicine, London, UK; West London Renal and Transplant Centre, Hammersmith Hospital, Imperial College Healthcare NHS Trust, London, UK; Department of Haematology, Hammersmith Hospital, Imperial College Healthcare NHS Trust, London, UK; Hugh and Josseline Langmuir Centre for Myeloma Research, Centre for Haematology, Department of Immunology and Inflammation, Imperial College London, UK; Imperial College, Centre for Inflammatory Disease, Dept Immunology and Inflammation, Faculty of Medicine, London, UK; North West London Pathology, Charing Cross Hospital, London, UK

**Keywords:** chronic kidney disease, Fanconi syndrome, monoclonal gammopathy of renal significance, multiple myeloma, proximal tubular cells

## Abstract

**Background:**

Light chain proximal tubulopathy (LCPT) is a rare form of paraprotein-related disease, occurring in two main histopathological forms: crystalline and non-crystalline. The clinicopathological features, treatment strategies and outcomes, especially of the non-crystalline form, are not well described.

**Methods:**

We conducted a single-centre retrospective case series of 12 LCPT patients, 5 crystalline and 7 non-crystalline, between 2005 and 2021.

**Results:**

The median age was 69.5 years (range 47–80). Ten patients presented with CKD and significant proteinuria (median estimated glomerular filtration rate of 43.5 ml/min/1.73 m^2^; urine protein:creatinine ratio 328 mg/mmol). Only six patients had known haematological disease at the time of renal biopsy. Multiple myeloma (MM) was diagnosed in seven patients cases and monoclonal gammopathy of renal significance (MGRS) in five patients. A clone was detected in all cases combining serum/urine electrophoresis and free light chain (LC) assays. Crystalline and non-crystalline variants had similar clinical presentations. For the non-crystalline variant, a diagnosis was reached based on a combination of CKD without another cause, haematological workup, LC restriction on immunofluorescence and abnormalities on electron microscopy (EM). Nine of 12 patients received clone-directed treatment. Patients who achieved haematological response (including all non-crystalline LCPT) had improved renal outcomes over a median follow-up of 79 months.

**Conclusions:**

The non-crystalline variant may go unrecognised because of its subtle histopathological features and requires EM to distinguish it from ‘excessive LC resorption without tubular injury’. Clone-directed treatment with good haematological response improves renal outcomes in both variants but limited data exist in MGRS. Multicentre prospective studies are needed to better define the clinicopathological characteristics associated with poor outcomes and optimize treatment strategies in patients with MGRS.

KEY LEARNING POINTS **What is already known about this subject?**

- Light chain proximal tubulopathy (LCPT) is rare and presents in two main histopathological forms: crystalline and non-crystalline.
- The clinicopathological characteristics, outcomes and treatment strategies, especially in the non-crystalline forms, are not well described.

**What this study adds?**

- Haematological diagnosis is often unknown at the time of renal biopsy and it is the demonstration of LCPT on biopsy that leads to a haematological diagnosis. Most patients present with chronic kidney disease (CKD), indicating late diagnosis. Crystalline and non-crystalline forms have similar clinical presentations.
- Demonstration of light chain restriction required protease digestion of formalin-fixed, wax-embedded tissue in all of our cases. Light microscopy (LM) is often non-informative, examination of semi-thin toluidine blue–stained sections may be helpful, but electron microscopy (EM) is key, especially for non-crystalline variants.
- Progression to CKD is common but is often slow. Haematological clone-directed treatment improves outcomes in crystalline and non-crystalline cases, however, limited data exist for cases of LCPT seen specifically in the context of monoclonal gammopathy of renal significance with regard to treatment and outcome.

**What impact this may have on practice or policy?**

- Promote a multidisciplinary integrative approach between renal histopathologists, nephrologists and haematologists.
- Increase familiarity with the very subtle LM findings of LCPT, the need for protease digestion to detect the light chains on immunofluorescence and the need for detailed examination of tubules on EM.
- Non-crystalline as well as crystalline forms are associated with poor outcomes, and optimal treatment strategies are not firmly established. Therefore, international and national collaborative registries are needed.

## INTRODUCTION

Monoclonal gammopathies can affect the kidney in myriad ways, involving the glomeruli, tubules, interstitium and blood vessels. In light chain proximal tubulopathies (LCPTs), reabsorption of excessive monoclonal free light chains (LCs) and accumulation within the proximal tubular epithelium leads to tubular dysfunction and activation of pro-inflammatory and profibrotic pathways. Certain amino acid sequences and structural conformations of the variable domain of LCs increase their potential to form crystals, rendering them nephrotoxic even at low concentrations [1].

LCPT can occur in two main histopathological forms [2–6]. In the crystalline form, the LCs (usually kappa) form electron-dense ‘crystalline structures’, usually rhomboid or ‘spicule’-like crystals, within lysosomes and/or the cytoplasm. In the non-crystalline form, three variants are described: amyloid proximal tubulopathy, LCPT without organised deposits and LCPT with large fibrillary aggregates. In the latter, tubular cytoplasm contains bundles of fibrils on electron microscopy (EM). In amyloid proximal tubulopathy, intracytoplasmic fibrils with typical features of amyloid, including Congo red positivity are seen. In LCPT without organised deposits, there is acute tubular injury, with light restriction on immunofluorescence (IF) but non-specific EM findings such as an increase in cytoplasmic lysosomes, which may include mottled appearances [7], although mottled lysosomes are not specific to light chain tubulopathy. LCPT without organised deposits poses a diagnostic challenge, as it needs to be distinguished from physiological trafficking of light chains within proximal tubular cells. Whereas a causative established haematological malignancy requires treatment, when the tubulopathy occurs in the context of monoclonal gammopathy of renal significance (MGRS), the therapeutic approach is based on small case series and expert opinion. In particular, the treatment approach and outcomes in non-crystalline forms, especially in the context of MGRS, has not been systematically described or studied in large case series and compared with the crystalline variant.

We report our experience with LCPT over the last 16 years (2005–2020), with a focus on clinical and pathological findings in the non-crystalline cases. We also review the literature on LCPT and summarise the clinicopathological correlations.

## MATERIALS AND METHODS

We reviewed the clinical records and biopsy database in our centre for cases of crystalline and non-crystalline LCPT, with the objective of defining the morphological and clinical features of cases diagnosed during 2005–2020 with follow-up until August 2022. Definitions and biopsy processing techniques are summarised in the supplementary material.

## RESULTS

### Kidney biopsy histology

Twelve biopsies with LCPT were found in the database of 6493 native kidney biopsies performed in our centre, representing 0.18% between 2005 and 2020 (Table 1). Seven patients had non-crystalline LCPT and five patients had crystalline LCPT. Details of histological findings are provided in Table 1.

**Table 1:** Histopathology findings in our 12 cases.

	LM					IF				EM	
Variant	Quality of tubular cytoplasm	IFTA, %	Other tubular findings	Glom sclerosed, %	Glomerular findings	IF glomeruli frozen	Gloms FFPE	Tubules frozen light chains	Tubules FFPE	EM glomerular appearance	Appearance
1, non-crystalline	Plump cytoplasm (diffuse); large clear vacuoles on H&E and TB	10	n/a	4.54	Increased	Linear kappa	Negative	Kappa = lambda	Kappa restricted—linear TBM + granular cytoplasmic	2 glom FSGS	Fibrils (randomly arrayed) + fine granules in membrane-bound structures and in cytoplasm
2, non-crystalline	Shaggy cytoplasm; large pale cytoplasmic vacuoles on H&E and TB	5	Interstitial oedema; tubular cholesterol clefts	11.11	Normal	Not done	Negative	Kappa = lambda	Kappa restricted—granular cytoplasmic	slight GBM thickening	Fibrils (parallel stacks) in membrane-bound structures and in cytoplasm
3, non-crystalline	Plump cytoplasm (focal); fine and coarse clear vacuolation on H&E and TB	15	n/a	16.66	Normal	Not done	Mes IgM 1+	Not done	No restriction demonstrated	No glomeruli	Fibrils (some parallel, some random) + fine granules in membrane-bound structures
4, non-crystalline	Plump cytoplasm (diffuse); small clear vacuoles on H&E and TB	10	Patchy chronic inflammation	31.70	Ischaemia	Not done	Not done	Not done	Kappa restricted—granular cytoplasmic	Slight GBM thickening	Fibrillogranular, some ‘fingerprints’ in membrane-bound structures and in cytoplasm
5, non-crystalline	Acute tubular injury with a few clear cytoplasmic vacuoles on H&E and TB	20	n/a	30.77	Ischaemia; HT	Not done	Gr mes IgM 1 + IgA±	Kappa = lambda	Lambda predominance—granular cytoplasmic	Slight GBM thickening	Fibrillogranular (randomly arrayed)
6, non-crystalline	Plump cytoplasm (focal); eosinophilic granules on H&E; dark vacuoles on TB	80	Incidental papillary adenoma	43.47	3× FSGS, ischaemia, HT, increased MM	Linear CW IgG 1+; Gr Mes IgM±	Negative	Kappa = lambda	Kappa restricted—granular cytoplasmic	GBM thickening	Fibrillogranular (randomly arrayed) in membrane-bound structures
7, non-crystalline	Acute tubular injury NOS on H&E and TB	15	Patchy chronic inflammation	6.66	Normal	No glomeruli	Kappa = lambda	Kappa = lambda	Kappa predominance—granular cytoplasmic	Nothing of note	Electron densities in vacuoles, rare parallel fibrils
8, crystalline	Shaggy cytoplasm; rare irregular clear vacuoles on H&E; dark spicules on TB	30	Active TIN	33.33	Normal	Negative	Negative	Kappa = lambda	Kappa restricted—granular cytoplasmic	GBM thickening	Crystals; rhomboid/spicules (electron dense)
9, crystalline	Shaggy cytoplasm; crystals visible on H&E; dark spicules/rhomboids on TB	0	N/A	0	Normal	No glomeruli	Kappa = lambda	Kappa = lambda	Kappa restricted—granular cytoplasmic	Slight GBM thickening	Crystals; rhomboid (electron dense), with ‘grid’-like substructure
10, crystalline	Featureless cytoplasm on H&E; rare dark spicule on TB	0	N/A	0	Normal	No glomeruli	Negative	Kappa = lambda	Kappa restricted—granular cytoplasmic	Nothing of note	Crystals; rhomboid/spicules (electron dense)
11, crystalline	Plump cytoplasm (focal); featureless on H&E and TB	20	–	41.66	2× FSGS; crystalline podocytopathy	IgM/C3 in segmental scars	Kappa restricted crystals in podocytes	Kappa = lambda	Kappa restricted—crystals cytoplasmic	Slight GBM thickening; spicules in podocytes	Crystals; spicules (electron lucent)
12, crystalline	Featureless cytoplasm on H&E; rare clear spicule on TB	30	Interstitial lymphoplasmacytic infiltrate susp for lymphoma	30	Ischaemia	Mes IgM 1+	Mes IgM 1+	Kappa = lambda	Kappa restricted—crystals cytoplasmic	Nothing of note	Crystals; rhomboid/spicules (electron lucent)

H&E: haematoxylin and eosin; IFTA: interstitial fibrosis tubular atrophy; TIN: tubulointerstitial nephritis; FSGS: focal segmental glomerulosclerosis; Gr mes: granular mesangial; LM: light miscoscopy; IF: immunofluorescense, EM: electron microscopy; FFPE: formalin fixed paraffin embedded.

**Figure 1:**
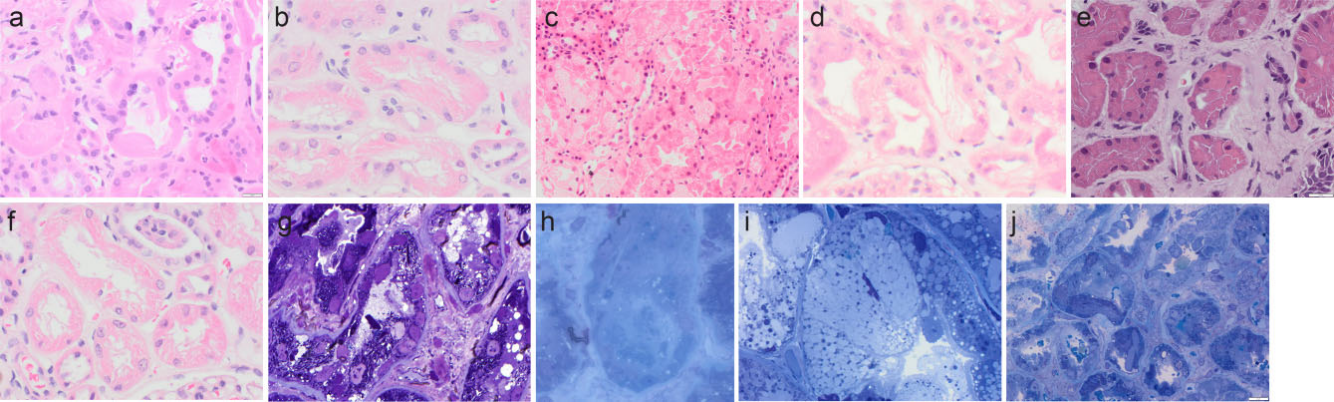
Light Microscopy features. 1a–f: H&E stains. (**a**) Focally “plump” proximal tubular epithelial cell cytoplasm (PTEC) (**b**) Diffusely “shaggy” cytoplasm in PTEC (**c**) Diffusely vacuolated cytoplasm in PTEC (**d**) Cytoplasmic clearing in PTEC (**e**) Eosinophilic vacuoles in PTEC (**f**) Rhomboid crystals in PTEC. 1g–j Semi-thin toluidine blue (TB)-stained sections of the samples for EM. (**g**) Dark rhomboid intracytoplasmic crystals; (**h**) Light/clear intracytoplasmic spicules; (**i**) Light intracytoplasmic rounded vacuoles; (**j**) Extensive areas of cytoplasmic clearing.

On light microscopy (LM), all samples showed evidence of acute tubular injury, often with large and/or small clear vacuoles in the cytoplasm. In many cases, the cytoplasm of proximal tubular epithelial cells appeared ‘plump’, ‘shaggy’ or vacuolated (Fig. 1a–f). Vacuolations were either focal or diffuse, mostly clear, but eosinophilic in one case. In one case, rhomboid intracytoplasmic crystals were visible on standard LM examination (Fig. 1g). Examination of semi-thin toluidine blue (TB)-stained sections of the samples for EM revealed rhomboid and/or spicular crystals in four of the five crystalline variant cases, although in some cases these were scarce (Fig. 1g, h). In non-crystalline cases, TB-stained semi-thin sections showed light cytoplasmic vacuoles, sometimes abundant (Fig. 1i), or extensive areas of cytoplasmic clearing (Fig. 1j). All cases were negative for Congo red stain.

Other LM findings were limited; two cases had focal segmental glomerulosclerosis (FSGS) and one had active tubulointerstital nephritis (TIN).

**Figure 2:**
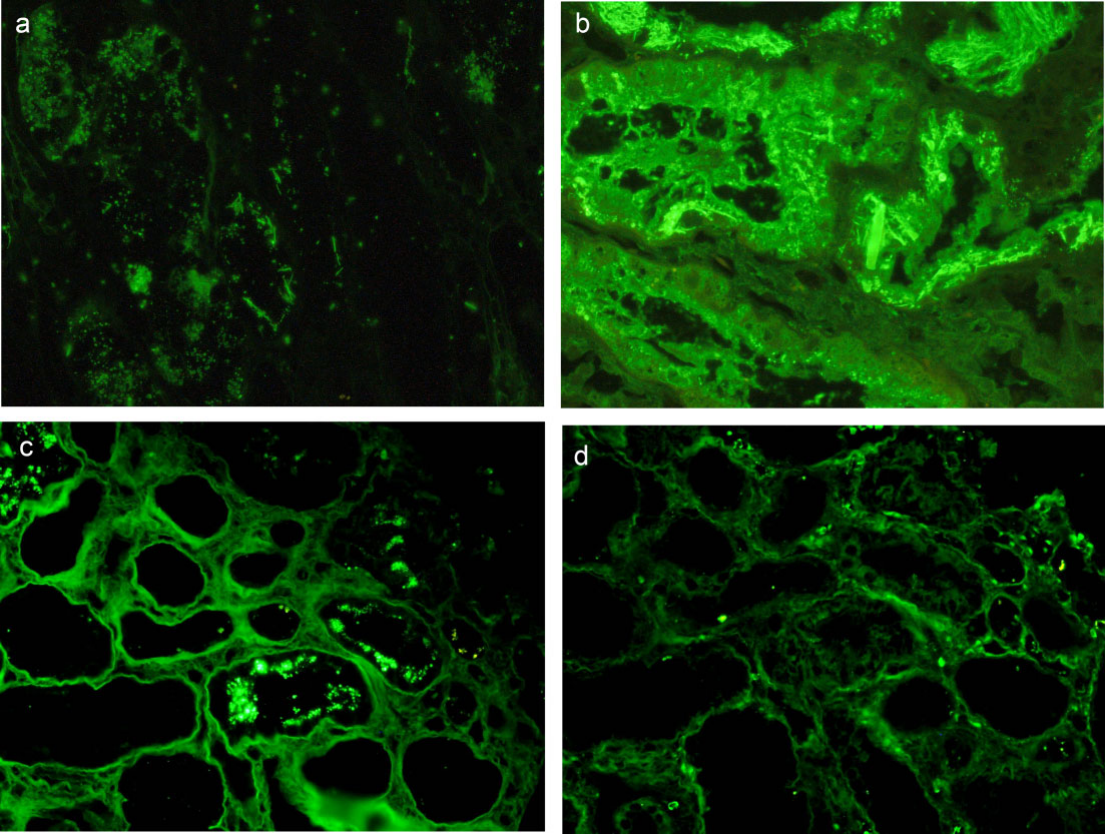
Immunofluorescence features (**a**) Kappa light chain-positive intracytoplasmic spicules; (**b**) Kappa light chain-positive intracytoplasmic rhomboid crytals; (**c**+**d**) Non-crystalline variant with granular preponderance of kappa (**c**) light chain over lambda (**d**) light chain.

On IF, light chain staining performed on frozen tissue was consistently of equal intensity without obvious restriction. Light chain IF was repeated on paraffin sections after protease digestion, where there was kappa light chain restriction in nine cases; in one case there was kappa predominance and in another there was lambda predominance. In crystalline cases, the IF showed rhomboid or linear ‘spicular’ features (Fig. 2a, b), whereas for the non-crystalline variant, there was a granular restriction or clear preponderance of one of the light chains (Fig. 2c, d).

**Figure 3:**
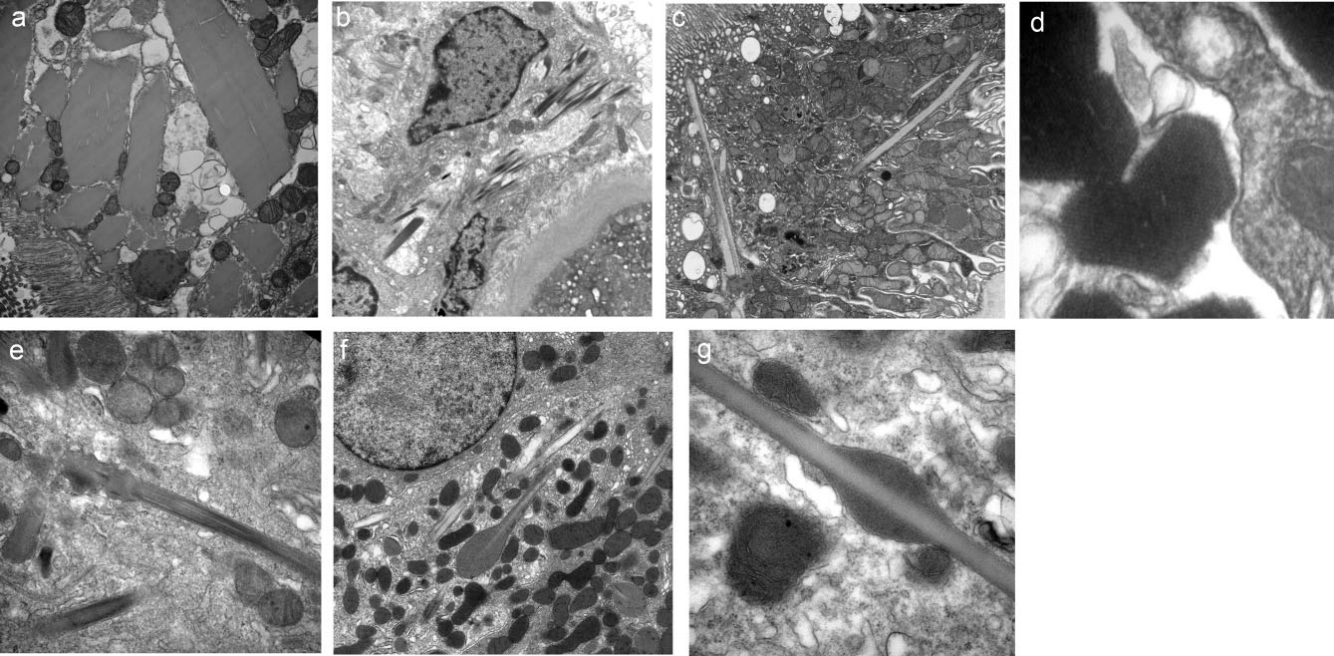
Electron Microscopy features, crystalline variant. Variety of appearances. (**a**) Case with electron dense rhomboid intra-cytoplasmic crystals in PTEC; (**b**) Case with interstitial macrophage containing electron dense spicules (**c**) Case with electron lucent spicules in PTEC; (**d**) High magnification showing a grid-like pattern, with 2 sets of parallel lines crossing in near-orthogonal directions in a case with electron dense intracytoplasmic rhomboid crystals; (**e**) Electron dense intra-cytoplamic elongated spicules with terminal swelling; (**f**) Electron dense and lucent intra-cytoplasmic elongated spicules, one with a terminal swelling giving it a “cotton ear-bud” appearance; (**g**) Lightly electron dense elongated spicule with a darker central swelling.

**Figure 4:**
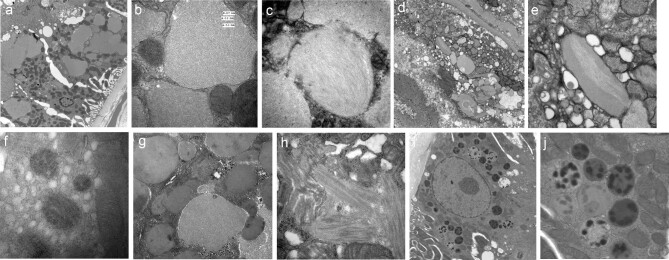
Electron Microscopy features, non-crystalline variant. Variety of appearances. (**a**) Low power, membrane-bound intracytoplasmic vacuoles; (**b**) Same case as (**a**). Membrane-bound vacuoles containing parallel fibrils; (**c**) Case with membrane-bound vacuoles containing randomly arranged fibrils; (**d**) Membrane-bound structures containing light and dark granulofibrillar material; (**e**) Same case as (**d**), higher magnification; (**f**) Case with intra-cytoplasmic finger-print-like structures; (**g**) Case with membrane-bound structures containing granular material; (**h**) Case with bundles on intracytoplasmic fibrils intersecting at variable angles; (**i**+**j**) Case with lysosomal dark “mottled” granules at low (**i**) and high (**j**) power; this appearance can be seen with a number of tubular insults and is not specific to LCPT.

On EM, cases of crystalline variant showed rhomboid structures or spicules lying freely in the cytoplasm or, for some, in membrane-bound structures, with variable degrees of electron density, some quite lucent (Fig. 3a–c). Most of these had no substructure, although one case on high magnification showed a grid-like pattern (Fig. 3d). In several cases the spicules had swellings, giving them an ‘cotton ear-bud’-like appearance (Fig. 3e–g). Non-crystalline variants mainly showed the presence of fibrils, often admixed with amorphous electron dense granular material, either in the cytoplasm and/or in membrane-bound structures. The appearances were remarkably variable (Fig. 4a–j): bundles of parallel fibrils, bundles of fibrils intersecting at different angles or randomly arrayed or cross-hatched fibrils. One case had intracytoplasmic finger-print-like structures; another case had abnormal lysosomal inclusions.

### Clinical characteristics

Three patients were female and nine patients were male. The median age was 69.5 years (range 47–80). Seven patients were on antihypertensive agents and none of the patients had diabetes at diagnosis. The median estimated glomerular filtration rate (eGFR) at the time of kidney biopsy was 43.5 ml/min/1.73 m^2^ [range 20–78, interquartile range (IQR) 33–60]. The median urine protein:creatinine ratio (UPCR) was 328 mg/mmol (range 0–819, IQR 85.5–746.5). Only two patients had an eGFR >60 ml/min/1.73 m^2^ at the time of biopsy; the rest of the patients had chronic kidney disease (CKD). Six patients had proteinuria in the nephrotic range but none of the patients presented with features of nephrotic syndrome. Two patients had no proteinuria. Fanconi syndrome (FS) was incompletely documented or tested in all cases. Three cases had normoglycaemic glycosuria, hypophosphatemia and hypouricemia and five patients had none of these signs.

Eight patients had a detectable paraprotein with serum protein electrophoresis (SPEP) and immunofixation electrophoresis (IFE) and all patients who had serum free light (SFLC) ratio tested had an abnormal result (*n* = 10). Urine protein electrophoresis (UPEP) and IFE detected the paraprotein in 11 of 12 patients. The underlying haematological diagnosis was multiple myeloma (MM) in six patients and MGRS in six patients [four had smouldering myeloma (SMM), one had non-Hodgkin lymphoma and one had asymptomatic Waldenström macroglobulinaemia (WM)]. In six patients the kidney biopsy was the initial invasive investigation that led to the haematological diagnosis with further work-up following, including bone marrow biopsies [bone marrow adipose tissue (BMAT)] and skeletal surveys. The remaining six patients had an established haematological diagnosis and were referred for a kidney biopsy to determine renal involvement due to eGFR changes or proteinuria. The majority of these patients were diagnosed with MGRS (five of six patients) (Table 2).

**Table 2:** Clinical characteristics in our 12 patients.

Characteristics	All cases	Non-crystalline	Crystalline
*N*	12	7	5
Age (years), median (range)	69.5 (47–80)	68 (57–80)	70 (47–75)
Sex	3 F/9 M	2 F/5 M	1 F/4 M
Renal disease (eGFR)	43.5 ml/min/1.73 m^2^ (range 20–78, IQR 33–60) 10/12 eGFR ≤60 ml/min/1.73 m^2^	36 ml/min/1.73 m^2^ (range 27–70, IQR 32–60) 6/7 eGFR ≤60 ml/min/1.73 m^2^	49 ml/min/1.73 m^2^ (range 20–78) 4/5 eGFR ≤60 ml/min/1.73 m^2^
Proteinuria (UPCR)	328 mg/mmol (range 0–819, IQR 85.5–746.5)	457 mg/mmol (range 9–819, IQR 280.5–776.5)	85 mg/mmol (range 0–759)
Prior haem diagnosis	6/12	3/7	4/5
Paraprotein	5 IgG kappa	3 IgG kappa	2 IgG kappa
	4 kappa only	2 kappa only	2 kappa only
	2 IgM kappa	1 IgM kappa	1 IgM kappa
	1 IgA lambda	1 IgA lambda	
Light chain	10 kappa	6 kappa	4 kappa
	1 lambda	1 lambda	
Urine paraprotein	11/12	6/7	5/5
SFLC	Abnormal 10/10	Abnormal 6/6	Abnormal 4/4
Haem diagnosis	7 MM	4 MM	3 MM
	5 MGRS (4 SMM, 1 lymphoma)	3 MGRS (2 SMM, 1 lymphoma)	2 MGRS (2 SMM)
Treatment	5 chemo	3 chemo	2 chemo
	4 chemo + ASCT	2 chemo + ASCT	2 chemo + ASCT
	2 conservative	2 conservative	1 n/a
	1 n/a		
Follow-up (months), median	79 (range 17–129, IQR 51.5–104)	79 (range 17–129)	78.5 (range 42–113)
Outcome	eGFR improvement (>10% eGFR change) or stabilisation (±10% eGFR change) in all treated patients 9/9 proteinuria improvement (>30% UPCR change) in all treated patients	eGFR improvement in all treated patients, worsening eGFR in 2 untreated, proteinuria improvement in all treated patients	eGFR improvement or stabilisation in all treated patients, 4/4 proteinuria improvement in all treated patients

UPCR: urine protein to creatinine ratio; MM: multiple myeloma; MGRS: monoclonal gammopathy of renal significance; SMM: smouldering myeloma; ASCT: autologous stem cell transplant; eGFR: estimated glomerular filtration rate(CKD-EPI).

In the non-crystalline LCPT subgroup (*n* = 7), the median eGFR at the time of biopsy was 36 ml/min/1.73 m^2^ (range 27–70, IQR 32–60) and UPCR was 457 mg/mmol (range 9–819, IQR 280.5–776.5). The underlying haematological diagnosis was MM in four patients and MGRS in three patients (two with SMM and one with non-Hodgkin lymphoma). Five patients had a detectable paraprotein with SPEP [three had an immunoglobulin G (IgG) kappa, one IgM kappa and one IgA lambda]. Six patients had detectable paraprotein with UPEP and all patients tested with an SFLC ratio had an abnormal result.

In the crystalline LCPT subgroup, the median eGFR and UPCR at presentation were 49 ml/min/1.73 m^2^ and 85 mg/mmol, respectively. Two patients were diagnosed with MM and three with MGRS (two with SMM and one with WM). Three patients had a detectable paraprotein with SPEP/IFE and all patients had a detectable paraprotein with UPEP/IFE.

### Follow-up

Nine patients received clone-directed treatment and two patients had conservative management on the basis of age and frailty (data not available for one patient). Four of the nine patients who received clone-directed treatment underwent melphalan-conditioned autologous stem cell transplant (ASCT). Four patients received bortezomib-based chemotherapy. The median follow-up was 79 months (range 17–129, IQR 51.5–104). Eight patients achieved complete response (CR) or very good partial response (VGPR) and one patient achieved partial response. All patients who responded to clone-directed treatment improved or stabilised their kidney function and proteinuria (Table 2, Supplementary Table S1). The two patients who did not receive clone-directed treatment and the one patient who did not respond to treatment had CKD progression (Supplementary Table S1).

Five of the seven patients with non-crystalline LCPT who received clone-directed treatment had CR or VGPR and all of them had improved renal outcomes. The underlying haematological diagnosis in these patients was MM in four patients and lymphoma in one. The two patients with non-crystalline LCPT who did not receive treatment were diagnosed with SMM and had CKD and proteinuria progression.

### Literature review

We identified a total of 336 cases of LCPT with or without FS published in the form of case reports or case series since 2000. The median age at presentation was 60.5 years (range 23–89). The median eGFR was 36.4 ml/min/1.73 m^2^ (IQR 4.3–161.1). CKD was common at presentation [196/272 (72%) with available data]. A total of 112/201 patients (56%) with available eGFR at presentation had an eGFR <60 ml/min/1.73 m^2^, with 60 of these patients having an eGFR <30 ml/min/1.73 m^2^. Proteinuria was a common finding, with 71/173 patients (41%) presenting with nephrotic range proteinuria (>3 g/day) and 98/173 (57%) presenting with proteinuria in the subnephrotic range. FS (full blown or incomplete) was present in 195/263 patients (74%) in the studies where investigations for elements of FS were performed. Serum protein electrophoresis was reported in 225/336 cases: IgG kappa was the most common paraprotein, identified in 83/225 (37%), followed by kappa only LC in 60/225 (27%). Overall, the underlying haematological diagnosis was MM in 109 patients, overt WM in 4, SMM in 53, MGUS in 119, plasmacytoma in 1 and low-grade lymphoproliferative disease in 13 (1 CLL, 6 lymphoma and 6 WM). Based on the International Kidney and Monoclonal Gammopathy research group consensus criteria, 200/313 patients (64%) could be reclassified as MGRS and 113 (36%) as MM (and 4 overt MW).

Renal histopathology was available in 286/336 patients. Thirteen patients had concomitant cast nephropathy, 19 had concomitant podocytopathy and 15 had crystal-storing histiocytosis (CSH). Crystalline LCPT was described in 193 patients (69%) and non-crystalline forms of LCPT were described in 83 (31%) (Supplementary Table S2). Five were described as amyloid proximal tubulopathy.

Individual subject data from our literature review with sufficient clinicopathological correlations were available for 150 patients (103 crystalline and 47 non-crystalline LCPT). Of the 47 patients with non-crystalline LCPT, nephrotic syndrome was reported in 21/36, CKD in 20/31 and FS in 12/24. The involved LC was kappa in 18 and lambda in 22 (7 not available). The underlying haematological diagnosis in non-crystalline forms was MM in 26 and MGRS in 16. In the 107 patients with crystalline LCPT with available clinical data, nephrotic range proteinuria was reported in 33/95, CKD in 40/53 and FS in 45/91. The LC involved was kappa in 88/92 and lambda in 5/92. The haematological diagnosis was MM in 43 and MGRS in 61.

Treatment regimens and outcomes were heterogeneous and are summarised in Table 3 and the Supplementary data. Since 2000, six retrospective cohort studies with 10–49 FS patients have described the haematological diagnosis, treatment and outcomes (Table 3) [4, 8–12]. Of note, renal biopsies were not performed in all patients in these cohort studies. The most recent studies demonstrated stabilisation or improvement of renal function, as well as tubular response in some cases (resolution of FS) with the use of newer antimyeloma agents and/or ASCT. Renal response was dependent on the depth of the haematological response [4, 10–12]. Only one study from 2004 (from a single institution, covering 1968–2002) showed a significant risk of complications with the use of older alkylating agents outweighing the benefits on kidney function [9]. All studies included patients with MM and MGRS and described crystalline and non-crystalline LCPT variants in the treatment group, but separate analysis was not possible. Progression to end-stage kidney disease (ESKD) was slow in all patients who did not receive treatment in these six cohorts.

**Table 3:** LCPT case series with outcomes.

Characteristics	Messiaen 2000, France	Ma 2004, USA	Stokes 2016, USA	Vignon 2017, USA/France	Wu 2019, China	Chen 2021, China
N	11	32	46	49	22	26
Age (years), median (IQR)	65 (42–86)	58 (31–81)	60 (39–87)	58 (37–84)	49 (30–76)	54.7 (±14.7)
Sex	6 F/5 M	10 F/22 M	16 F/30 M	19 F/30 M	14 F/8 M	15 F/11 M
Renal disease	11/11 CKD	SCr 176.8 μM (79.56–327.08)	eGFR 36.4 ml/min	SCr 171 μmol/min	SCr 93 μmol/l	SCr 91 μmol/l
			83% CKD	(70–1278)	(38–348)	(38–270 μmol/l)
			22% AKI (baseline SCr not available in all)	eGFR 33 ml/min (4–111) 46/49 CKD	eGFR 66 ml/min (13–126 ml/min) 7/22	eGFR 68 ml/min (±26.4)11/26 eGFR <60 ml/min
					eGFR <60 ml/min	
Proteinuria	3/11 nephrotic range8/11 subnephrotic	NA	17/46 nephrotic range29 subnephrotic (6/29 <1 g/day and 23 patients with 1–3 g/day)	All patients had proteinuria >0.5 g/dayMedian 1.5 g/day (IQR 0.5–10)	3.1 g/day (IQR 0.1–10.3)	2.34 g/day (IQR 0.08–13.76)
Fanconi syndrome	11/11	32/32	17/45 FS	All patients with at least on sign of proximal tubulopathy17 patients full-blown FS	17/22 full-blown FS5/22 at least one sign of proximal tubulopathy	13/26 full blown FS
Prior haem diagnosis	2/11 MGUS 9/11 no haem dx	NA	7/46	NA	NA	NA
Renal biopsies	11/11	17/32	46/46	39/49	10/22	10/26
Renal biopsy findings	8/11 crystalline	8/17 crystalline	40/46 crystalline	24/39 crystalline	No crystalline inclusions by LM	6/10 (?)
	3/11 non-crystalline3 concomitant MCN	9 non-crystalline	6/46 non-crystallineDetection IF-frozen 15/43Detection IF-pronase 37/383/46 CSH3/46 podocyte inclusions	15/39 non-crystalline3 concomitant LCDD3 concomitant MCN6 had CSH:1 on renal biopsy1 in pleural fluid1 femur biopsy		
Paraprotein (SPEP+/-IFE)	2/11 IgG kappa	9/32 IgG kappa	17/46 IgG kappa	21/49 IgG kappa	6/22 IgG kappa	7/26 IgG
	2/11 IgA kappa	3/32 IgA kappa	4/46 IgA kappa	2/49 IgG lambda	2/22 IgA kappa	2/26 IgA
	6/11 kappa	2/32 IgM kappa	2/46 IgG lambda	16/49 kappa	2/22 IgA lambda	1/26 IgM
	1 no paraprotein	5/32 kappa	10/46 kappa	4 IgM lambda	1/22 IgM kappa	16/26 light chain only
		3/32 IgG lambda	12/46 negative	5 IgA kappa	11/22 kappa	
		10/32 negative	1/46 unknown	1 IgA lambda		
Light chain	11/11 kappa	29/32 kappa	43/45 kappa	46/49 kappa	20/22 kappa	22/26 kappa
		3/32 lambda	2/45 lambda	3/49 lambda	2/22 lambda	4/26 lambda
			1/45 no stain, kappa in urine			
Urine paraprotein (UPEP+/-IFE)	1IgG kappa	29/32 kappa	25/46 kappa	All patients had detectable urine monoclonal LC	N/A	Detected in 18 of 20 tested
	1 IgA kappa	3/32 lambda	5/46 IgG kappa			
	9/11 kappa		1/46 IgA kappa			
			2/46 negative			
			13/46 unknown			
SFLC	N/A			Abnormal in 34 studied cases	N/A	Abnormal in 10 out of 10 tested
Haem. diagnosis	8 MGRS	20 MGRS	15 MM	7 MM	6 MM	10 MM
	(6 MGUS, 2SMM)3 MM	(14 MGUS, 6 SMM)10 MM2 WM	21 MGRS (21 MGUS, 7 SMM, 2 non-Hodgkin lymphoma, 1 CLL)	4 overt WM	16 MGRS	14 MGRS 1 WM
				38 MGRS (25 SMM, 13 MGUS)	(13 MGUS, 2SMM, 1WM)	1 primary plasma cell leukaemia (PPCL)
Treatment	5 no chemotherapy 6 had treatment (*old regimens)	22/32 received chemo	*Follow-up data for 3616/36 chemo + ASCT11/36 chemo9/36 none	7 no treatment (all MGRS)42 treatment	10 no treatment (1 MM, 1 SMM, 8 MGUS)12 had treatment (5 MM, 5 MGUS, 1 SMM, 1 WM)	13 had treatment (8 MM, 3 MGRS, 1 WM, 1 PPCL)
Follow-up (months)	12 (0–120)	65 (2–238)	39 (1–141) cryst. LCPT	45.6 (12–300)	37 (1–96)	36 (0–133)
			14.5 (5–24) non-cryst. LCPT			
Outcome	1/11 early death (no treatment)4/11 no treatment:– 1 death/CKD progress,– 2 CKD progression,– 1 stable CKD6/11 treatment– 4 CKD progression,– 1 death, stable CKD,– 1 no progression	14 deaths (7 MM, 4 MGUS, 2 SMM)5/32 progressed to ESKD (1 MM, 4MGUS)No change in eGFR in patients MGUS, SMM receiving chemo (data not available)	30 crystalline LCPT22/30 received treatment (4 deaths, 18 stable or improved CKD, 4 CKD progression)8/30 no treatment,– 3 deaths,– 5 stable or improved CKD,– 2 ESRD6 non-crystalline LCPT1/6 presented with ESRD1/6 untreated—stable normal function 10-month follow-up4/6 treated patients:– 3/4 stable CKD,– 1/4 normal function resolution of proteinuria	9 deaths due to haem progression3 deaths in ESRD with sepsis (2 with secondary myelodysplastic)2 deaths of unknown cause7 no treatment:4/7 ESRD (median 15 years)3/7 stable CKD (median 3 years)42 had treatment37/42 haem response with renal stabilization5 ESRD (2 MCN, 1 severe IFTA, 2 multiple chemo regimens)13/38 renal tubular function improvement (11/13 achieved VGPR haem response)	3 deaths (progression of MM, cerebral haemorrhage, unknown cause)Significant improvement in eGFR in patients receiving chemotherapyPatients on bortezomib-based regimens had better haem response and tubular function response	4/26 deathsNo ESRDTreated patients had significant improvement in eGFR, proteinuria and FSAt 36 months eGFRTreatment versus non-treatment(81.1 ± 15.0 versus 54.6 ± 10.7 ml/min/1.73 m^2^, *P* = .032)

CKD: chronic kidney disease; MCN:myeloma cast nephropathy; MGUS: monoclonal gammopathy of undetermined significance; AKI: acute kidney injury; SPEP: serum protein electrophoresis; IFE: immunofixation; CSH: crystal-storing histocytosis; LCDD: light chain deposition disease; FS: fanconi syndrome.

## DISCUSSION

We made several important observations pertaining to both clinical and histological findings: haematological diagnosis is often unknown at the time of renal biopsy and it is the demonstration of LCPT on biopsy that leads to a haematological diagnosis; demonstration of light chain restriction required protease digestion of formalin-fixed, wax-embedded tissue in all of our cases; LM is often non-informative, examination of semi-thin TB-stained sections may be helpful, but electron microscopy is key, especially for non-crystalline variants; non-crystalline variants are mostly ‘fibrillar’ or ‘fibrillogranular’ in type, with a wide range of appearances; progression to CKD is common but is often slow; haematological clone-directed treatment improves outcomes in crystalline and non-crystalline cases, however, limited data exist for cases of LCPT seen specifically in the context of MGRS with regard to treatment and outcome.

Patients with LCPT present commonly in the sixth decade of life, when diabetes and hypertension are highly prevalent. The presenting features are CKD and significant proteinuria, often in the nephrotic range but without overt nephrotic syndrome. The fact that the majority of cases have established CKD at the time of renal biopsy indicates the slowly progressive nature of this entity and the need for earlier diagnosis. A small number of instructive cases, including one of our own, were LCPT post-transplantation, prompting retrospective review of the native biopsies with ancillary techniques, demonstrating evidence of LCPT as the cause of ESKD [13–17]. Biological evidence of tubular dysfunction with FS (full blown or incomplete) is commonly described in association with LCPT. However, based on our literature review, not all LCPT will have features of FS, especially in the non-crystalline variants. It is therefore important to distinguish LCPT with evidence of tubular dysfunction based on the presence of FS, as eloquently shown in a case series by Vignon *et al*. [10]. Moreover, FS was not formally investigated in a large proportion of cases, including our own case series, indicating a need for increased awareness and better characterization of the clinical consequences (i.e. for skeletal disease and CKD progression) in those cases where tubular dysfunction is present.

Strikingly, the haematological diagnosis is often unknown at the time of renal biopsy and it is the renal biopsy diagnosis that triggers investigations with BMAT and imaging to reach a haematological diagnosis. This highlights the important role for the renal pathologist in having a high degree of suspicion for this diagnosis in patients with unexplained CKD and proteinuria. Eventually, >50% of cases will have an MGRS as the underlying haematological diagnosis, as opposed to an overt malignancy like MM or symptomatic WM and lymphomas. Accurate haematological diagnosis has significant implications on the therapeutic strategies, especially in older patients. The paraprotein could be detected in all cases using a combination of SPEP, UPEP, IFE and SFLC assays. Even in the rare cases with negative SPEP, the involved paraprotein could be identified with UPEP and SFLC. The most common involved LC is kappa; however, lambda LC was described in ≈30% of cases in our literature review.

Non-crystalline forms of LCPT present with similar clinical features to crystalline forms. Indolent CKD and proteinuria are the main findings at the time of renal biopsy. FS is described in crystalline and non-crystalline forms and therefore it should be part of the workup in all LCPT. A clone is invariably detectable, either in the context of MGRS or overt haematological malignancy. Kappa LC involvement is more common in both crystalline and non-crystalline forms in our patients.

Previous reports have documented that demonstration of light chain restriction in crystalline tubulopathy may require IF on paraffin sections after protease digestion, which is needed to unmask antigens [18]. In our case series, this was also necessary for the non-crystalline variant. Previous reports indicate that in LCPT, tubular epithelial cells may show abundant ‘plump’ and homogeneous cytoplasm. We found that this feature could in some cases be very focal, limited to only some tubules and some cells within the tubule. We also noted cases with a ‘shaggy’ cytoplasm and cytoplasm vacuolations, that could be small or large, clear or eosinophilic. Although we had six cases of non-crystalline variant, none showed Congo red positivity (i.e. no cases of amyloid proximal tubulopathy).

An important observation in our study was the variety of appearances on EM in cases of non-crystalline LCPT [19–22]. A proportion of non-amyloid cases described in the literature are referred to as light chain proximal tubulopathy ‘with large fibrillary aggregates’. In our cases, the appearances were different for just about every case: fibrils were arranged in parallel bundles or randomly, lying freely in the cytoplasm or within cytoplasmic vacuoles [19, 23]. Some had a ‘cross-hatched’ appearance, with alternating fascicles in different directions, whereas in others the fibrils were laid out in neat bundles. In addition, we found that abundance was variable: in some cases, changes were noted in most tubules, but in others the changes were focal, requiring examination of a large number of tubules on EM to find convincing abnormalities. In non-crystalline tubulopathy without fibrils, there may be an increase in cytoplasmic lysosomes, which can be striking, or lysosomes may have abnormal inclusions and/or a mottled appearance [3, 7]. In association with a clinical picture of tubular dysfunction and monoclonal urinary light chains, such findings support a diagnosis of non-fibrillar LCPT. In our series, there was only one such case, the majority showed fibrillar or fibrillogranular inclusions.

When using EM to diagnose LCPT, care has to be taken to not consider other intratubular fibrils and inclusions, such as bundles of intermediate filaments and dysmorphic mitochondria. It is also worth noting that increased cytoplasmic vacuoles, including some with a mottled appearance, can be seen in association with many causes of tubular injury, so particular care should be taken (including clinical and IF findings) before attributing these to LCPT.

The rate of detecting serum monoclonal immunoglobulins in the context of monoclonal gammopathy of undetermined significance (MGUS) increases with age; up to 1.7% in those 50–60 years of age and increasing to 6.5% in those >80 years of age [24]. In these age groups, the prevalence of CKD, diabetes and hypertension is higher and a circulating paraprotein is often coexistent and not the cause of renal disease. In our literature review, only a small number of clinical studies have performed molecular studies to characterize LC sequences associated with FS [8, 25–28]. In the majority of the cases with pathogenic LC, the variable LC domains were derived from the *IGKV1-33* and *IGKV1-39* germline genes. Additionally, recent *in vitro* studies implicate specific FS-inducing LCs as a cause for morphological and functional changes in proximal tubular cells in low or physiologic concentrations regardless of their ability to form crystals. Certain molecular events were linked with FS-inducing LCs, but not in other LCs causing LCPT without FS [1]. These findings are in keeping with ours and others’ clinicopathological observations, describing LCPT as a protean entity presenting with a spectrum of clinical and histopathological manifestations.

Clone-directed treatment improved or stabilised renal function and/or proteinuria in our crystalline and non-crystalline cases, especially in those patients with CR or VGPR haematological response. The case mix included both MM and MGRS patients with a follow-up time greater than most of published studies, with a median of 73 months. A strength of our study is the detailed histopathologic description with clinical correlations. However, the small number and the lack of a comparison group for treatment is a limitation, as in all previous studies.

Our findings on LCPT outcomes are in keeping with other case series (Table 3). Early haematologic response is one of the most important factors for renal function preservation. However, in MGRS, the lack of prospective studies and the potential toxicity of antimyeloma regimens for single-organ, low-grade haematological disease is a concern for physicians. Bortezomib has a favourable renal profile and it is used without dose adjustment in CKD, but neuropathy is a common side effect. Other agents are increasingly being used in MGRS. Daratumumab, an anti-CD38 monoclonal antibody with proven efficacy in MM or AL amyloidosis, is of particular interest. In an open-label phase II trial in patients with proliferative glomerulonephritis with monoclonal immunoglobulin deposits, daratumumab had an acceptable safety profile and resulted in improvement of renal outcomes (NCT03095118). More recently, Kastritis *et al.* [29] described their experience with the use of daratumumab in a single-centre retrospective study of 25 patients with MGRS. Daratumumab-based regimens were used as a primary therapy for relapsing/refractory disease, showing improvement in proteinuria, eGFR stabilization and low toxicity. This study did not include patients with isolated LCPT.

Light chain tubulopathy is a rare disease, but increasingly recognized in the context of MGRS or overt haematological malignancy. The non-crystalline form has particularly protean ultrastructural features, such that attempts to further subclassify it may be futile. What is more important is to emphasise the need for familiarity with the very subtle LM findings, the need for protease digestion to detect the light chains on IF and the need to sometimes hunt in many tubules on EM to find the abnormal tubular epithelial cell inclusions. This is important because the non-crystalline and crystalline forms are associated with poor outcomes and optimal treatment strategies are not firmly established. Aggressive treatment of the clone may have benefit in cases with altered renal function, but the renal benefit needs to be balanced against the toxicity of treatment and infectious complications, especially in frail older patients and in those with advanced CKD. Importantly, limited data exist for non-crystalline forms with MGRS and multicentre prospective studies are needed.

## Supplementary Material

gfad085_Supplemental_File

## Data Availability

Data are available upon reasonable request.
