# Supplementary material for: Non-crystalline light chain proximal tubulopathy, a morphologically protean entity
Source: Nephrol Dial Transplant. 2023 Apr 29;38(11):2576–88. doi: 10.1093/ndt/gfad085 (PMC10615624; doi:10.1093/ndt/gfad085)
Supplement: gfad085_Supplemental_File [file gfad085_supplemental_file.docx]

**Supplementary Material**

Methods

*Patients*

*We have identified all patients with biopsy proven light chain proximal tubulopathy (LCPT) from the database of West London Renal and Transplant Centre, Imperial College NHS trust between 2005-2020 and follow-up until August 2022. All the renal biopsies were reviewed at the Imperial MGRS multidisciplinary meeting consisting of three renal histopathologists, three haematologists and four nephrologists caring for patients with paraprotein-related renal disease in West London. Subsequently, patients’ records were retrospectively reviewed and clinical and histopathological parameters were collected and analysed.*

*Renal Biopsy samples processing*

*All kidney biopsies were indication biopsies for renal dysfunction. Biopsies were split at bedside and placed into neutral buffered formalin for light microscopy (LM), Michel’s medium for processing to frozen for immunofluorescence (IF), and glutaraldehyde fixative for electron microscopy (EM). For LM, serial 1-2 micron sections were stained with haematoxilin and eosin, periodic acid Schiff, Jones methanime silver, Sirius red combined with elastin stain (von Gieson) and Congo red. IF was performed for IgG, IgA, IgM, C3, C1q, kappa and lambda clones. Where IF on frozen was negative or equivocal for light chains, it was repeated on paraffin section after pronase digestion. For EM, semi-thin toluidine blue-stained sections were first examined, then ultra-thin sections examined. LCPT was diagnosed when IF showed restricted staining for only one light chain within proximal tubular epithelial cells, and further classified into crystalline and non-crystalline variants. All crystalline variants had rhomboid or “spicule”-like crystals, within lysosomes and/or in the cytoplasm. Non-crystalline variant was diagnosed when no crystals were seen, but instead other ultrastructural abnormalities such as fibrils were noted, along with excess light chains in the urine and renal dysfunction without any other identified cause. Full details of non-crystalline variant cases are given in Results. Patients with cast nephropathy were excluded.*

*Definitions*

*MGRS definition was based on the 2019 International Kidney and Monoclonal Gammopathy Research Group consensus statement. Haematological treatment response definitions were based on the International Uniform Response Criteria to MM. Renal outcomes were defined based on change of proteinuria and eGFR. Stable eGFR was defined as ±10% change in eGFR between the eGFR on the day of the renal biopsy and at the end of follow-up. Improvement of eGFR was defined as > 10% positive change. We used CKD-EPI to calculate eGFR. Improvement of proteinuria was defined as > 30% reduction in uPCR between the uPCR on the day of the renal biopsy and at the end of follow-up.*

*Ethics*

*As a retrospective review the study met the criteria for service evaluation study for the establishment of Imperial MGRS MDT aiming to assess and expand service provision in West London; hence it not required approval from a research committee.*

*Literature review*

*For the literature review we have included case reports and case series published since 2000 in Medline, using custom designed search algorithms consisting of Medical Subject Headings (MeSH) terms, relevant short terms or combinations in the title or abstract. Reference lists of all selected studies were searched for additional studies. A list of included studies is presented in supplementary table 2 and all collected data are available on reasonable request in excel format.*

Supplementary Table 1: clinical characteristics, treatment and outcome

|  | **#** | **Renal diagnosis** | **Demographics** | | **Renal work-up** | | | | | | | | **Haematological work-up** | | | | | | **Treatment** | **Follow-up** | | | | |
| --- | --- | --- | --- | --- | --- | --- | --- | --- | --- | --- | --- | --- | --- | --- | --- | --- | --- | --- | --- | --- | --- | --- | --- | --- |
|  |  |  | **Age** | **Sex** | **BG** | **F** | **eGFR** | **uPCR** | **%GS** | **%TA/**  **IF** | **IF LC restriction in tubules** | **EM inclusions** | **Serum PP** | **Urine PP** | **FLC ratio** | **BMAT clone** | **Haem diagnosis** | **Diagnosis pre/post renal biopsy** |  | **Duration (months)** | **Alive** | **Change in eGFR %** | **Change in uPCR %** | **Haem.**  **response** |
| Non - Crystalline | 1 | Fibrillar non-crystalline | 66 | F | N |  | 60 | 819 | 4.6 | 10 | K | Y | IgG K | K FLC | - | Y | MM | Post | ASCT | 129 | Y | +25 | na | VGPR |
|  | 2 | Fibrillar non-crystalline | 72 | M | HTN | Y | 27 | 457 | 11.1 | 10 | K | Y | N | N | 481 | Y | MM | Post | Chemo  therapy | 111 | Y | +19 | -96%  (30) | CR |
|  | 3 | Fibrillar non-crystalline | 58 | F | N | N | 30 | 328 | 16.7 | 15 | N | Y | IgG K | K FLC | 782 | Y | MM | Post | ASCT | 97 | Y | +23 | -40%  (194) | VGPR |
|  | 4 | Fibrillar non-crystalline | 57 | M | N | Y | 36 | 9 | 31.7 | 15 | K | Y | N | K FLC | 362 | Y | MM | Post | Chemo  therapy | 79 | Y | +19 | 0 | VGPR |
|  | 5 | Fibrillar non-crystalline | 68 | M | HTN | - | 70 | 233 | 30.8 | 20 | L | Y | IgA L | IgA L  L FLC | 0.03 | Y | SMM | Pre | No treatment | 61 | Y | -16 | na | No change |
|  | 6 | Vacuolar non-crystalline | 80 | M | HTN  CVD  SMM | N | 34 | 819 | 43.5 | 80 | K | Y | IgG K | IgG K  K FLC | 12 | Y | SMM | Pre | No treatment | 30 | N | -74 | +15  (950) | No change |
|  | 7 | Vacuolar non-crystalline | 71 | M | HTN |  | 60 | 734 | 7.7 | 15 | K | Y | IgM K | IgM K  K FLC | 116 | Y | MGUS  (lymphoma) | Pre | Chemo  therapy | 17 | Y | 0 | na | VGPR |
| Crystalline | 8 | Crystalline | 74 | F | HTN |  | 20 | - | - | - | - | Y | N | K FLC | 343 | Y | MM | Post | Chemo  therapy | 113 | Y | +70 | na | VGPR |
|  | 9 | Crystalline | 69 | M | N | Y | 49 | 60 | 0 | 0 | K | Y | IgG K | K FLC | 230 | Y | MM | Pre | ASCT | 67 | Y | +63 | -75%  (25) | VGPR |
|  | 10 | Crystalline | 70 | M | N | N | 78 | 0 | 0 | 0 | K | Y | - | K FLC | - | Y | SMM | Pre | - | - | - | - | - | - |
|  | 11 | Crystalline | 47 | M | HTN | N | 54 | 759 | 41.7 | 20 | K | N | IgG K | IgG K  K FLC | 13 | Y | SMM | Pre | ASCT | 42 | Y | -10 | -53%  (356) | PR |
|  | 12 | Crystalline | 75 | M | CVD  HTN | N | 38 | 111 | 30 | 30 | K | Y | IgM K | IgM K  K FLC | 29 | Y | WM | Pre | DRC | 90 | Y | +23% | -90%  (11) | VGPR |

**%GS**, percentage glomerulosclerosis on renal biopsy by light microscopy; **%TA/IF**, percentage tubular atrophy and interstitial fibrosis on renal biopsy by light microscopy; **ASCT**, autologous stem cell transplant; add BG, explanation of abbreviation, **BMAT**, bone marrow aspiration and trephine; **CR**, complete response; **eGFR**, estimated glomerular filtration rate (expressed as mL/min/1.73m^2^, calculated by CKD-EPI); **EM**, renal biopsy electron microscopy; **FLC**, free light chains; **IF**, renal biopsy immunofluorescence; **Ig**, immunoglobulin; **K**, kappa; **L**, lambda; **LC**, light chain; **PP**, paraprotein; **uPCR**, urine protein to creatinine ratio (mg/mmol); **VGPR**, very good partial response.HTN, CVD,

Supplementary table 2: LCPT case reports and case series published after 2000

| **Study** | **Year** | **PMID** | **N** | **renal biopsies** | **Crystalline** | **Non-Crystalline** |
| --- | --- | --- | --- | --- | --- | --- |
| Messiaen | 2000 | 10844934 | 11 | 11 | 8 | 3 |
| Lajoie | 2000 | 11013962 | 1 | 1 | 1 | 0 |
| Minemura | 2001 | 11579959 | 1 | 1 | 1 | 0 |
| Decourt | 2003 | 12552516 | 1 | 1 | 0 | 1 |
| Bridoux | 2006 | 15806478 | 1 | 1 | 0 | 1 |
| Cai | 2006 | 16971357 | 1 | 1 | 1 | 0 |
| Nasr | 2006 | 16514437 | 1 | 1 | 1 | 0 |
| Kapur | 2007 | 17824791 | 5 | 5 | 2 | 3 |
| Taneda | 2009 | 18950910 | 1 | 1 | 0 | 1 |
| Said | 2006 | 16982631 | 1 | 1 | 1 | 0 |
| Balamuthusamy | 2009 | 19210683 | 1 | 1 | 1 | 0 |
| Larsen | 2011 | 21701535 | 13 | 13 | 3 | 10 |
| Rane | 2013 | 23523237 | 1 | 1 | 0 | 1 |
| Yao | 2014 | 24429451 | 1 | 1 | 0 | 1 |
| Akilesh | 2014 | 24439928 | 1 | 1 | 1 | 0 |
| Tomioka | 2004 | 15481856 | 1 | 1 | 1 | 0 |
| Elliot | 2010 | 19850387 | 1 | 1 | 1 | 0 |
| Ino | 2019 | 32734220 | 1 | 1 | 0 | 1 |
| Larsen | 2012 | 29497513 | 1 | 1 | 0 | 1 |
| Appleton | 2012 | 22612270 | 1 | 1 | 1 | 0 |
| Herlitz | 2009 | 19165173 | 1 | 1 | 0 | 1 |
| Kawamoto | 2019 | 30721454 | 1 | 1 | 0 | 1 |
| Gowda | 2015 | 25838654 | 1 | 1 | 0 | 1 |
| Matthai | 2020 | 32769341 | 1 | 1 | 1 | 0 |
| Boudhabhay | 2018 | 30593133 | 1 | 1 | 1 | 0 |
| Stompor | 2014 | 24512271 | 1 | 1 | 1 | 0 |
| Ryan | 2015 | 24849044 | 1 | 1 | 1 | 0 |
| Luciano | 2014 | 25295579 | 2 | 2 | 2 | 0 |
| Lerner | 2020 | 31907143 | 1 | 1 | 1 | 0 |
| Drieux | 2014 | 25492671 | 1 | 1 | 1 | 0 |
| Angioi | 2016 | 27321964 | 1 | 1 | 1 | 0 |
| Stehle | 2016 | 27367983 | 1 | 1 | 1 | 0 |
| Kishi | 2018 | 30101939 | 1 | 1 | 0 | 1 |
| Mathur | 2016 | 27424703 | 1 | 1 | 1 | 0 |
| Jung | 2020 | 32326898 | 5 | 5 | 2 | 3 |
| Brealy | 2018 | 30252563 | 1 | 1 | 0 | 1 |
| Gu | 2003 | 12673562 | 3 | 3 | 3 | 0 |
| Markowitz | 2000 | 10739805 | 1 | 1 | 1 | 0 |
| Stokes | 2006 | 16723980 | 1 | 1 | 1 | 0 |
| Figueres | 2015 | 25454480 | 1 | 1 | 1 | 0 |
| Yu | 2018 | 30419839 | 1 | 1 | 1 | 0 |
| Gallan | 2016 | 29142933 | 1 | 1 | 1 | 0 |
| Nishida | 2012 | 22385229 | 1 | 1 | 1 | 0 |
| Kurien | 2018 | 29497513 | 1 | 1 | 0 | 1 |
| Gibier | 2018 | 29052601 | 2 | 2 | 0 | 2 |
| Iliuta | 2016 | 27662203 | 1 | 1 | 0 | 1 |
| Linderman | 2021 | 33823814 | 1 | 1 | 1 | 0 |
| Duquesne | 2013 | 23313305 | 1 | 1 | 1 | 0 |
| Farooq | 2009 | 19119476 | 1 | 1 | 1 | 0 |
| El Hamel | 2010 | 20356978 | 3 | 3 | 3 | 0 |
| Liu | 2015 | 25791236 | 5 | 5 | 1 | 4 |
| Zakharova | 2017 | 25791236 | 1 | 1 | 0 | 1 |
| Keller | 2005 | 15705186 | 1 | 1 | 1 | 0 |
| Ungari | 2021 | 15705186 | 1 | 1 | 1 | 0 |
| Lin | 2020 | 32506206 | 1 | 1 | 1 | 0 |
| Chopra | 2020 | 32593151 | 1 | 1 | 1 | 0 |
| Jeon | 2015 | 26670310 | 1 | 1 | 1 | 0 |
| Lee | 2016 | 29957423 | 1 | 1 | 1 | 0 |
| Khalingi | 2017 | 2777521 | 1 | 1 | 1 | 0 |
| Ito | 2019 | 30702553 | 1 | 1 | 1 | 0 |
| Wang | 2016 | 26875962 | 1 | 1 | 1 | 0 |
| Patel | 2019 | 31799982 | 1 | 1 | 1 | 0 |
| Li | 2018 | 29035194 | 2 | 2 | 2 | 0 |
| Stokes | 2016 | 26374607 | 46 | 49 | 43 | 6 |
| Herrera | 2014 | 25268280 | 57 | 57 | 54 | 3 |
| Ma | 2004 | 15010372 | 32 | 17 | 8 | 9 |
| Wu | 2019 | 30511139 | 22 | 10 | 0 | 10 |
| Vignon | 2017 | 27435002 | 49 | 39 | 24 | 15 |
| Chen | 2021 | 33796233 | 26 | 10 | na | na |
| **TOTAL** |  |  | **336** | **286** | **193** | **83** |
